# Are explorers greener? Investigating the role of personality traits, connectedness to nature and attitudes toward exploring in various pro-environmental behaviors

**DOI:** 10.3389/fpsyg.2024.1404095

**Published:** 2025-01-15

**Authors:** Veronica Muffato, Laura Miola, Francesca Pazzaglia, Chiara Meneghetti

**Affiliations:** ^1^Department of General Psychology, University of Padua, Padua, Italy; ^2^Interuniversity Research Centre in Environmental Psychology (CIRPA), Rome, Italy

**Keywords:** pro-environmental behavior, personality traits, connectedness to nature, exploration, wayfinding

## Abstract

Previous research has suggested that individual characteristics, such as personality traits, are crucial for pro-environmental behaviors. However, the joint role of more specific environment-related individual dispositions on various pro-environmental behaviors has not yet been investigated and is the aim of this study. A total of 649 adults (18–59 years old) assessed their pro-environmental behaviors, personality traits, the connectedness to nature, attitudes toward exploration, and spatial anxiety. Personality traits (openness and conscientiousness) were related to some of the pro-environment behaviors (transportations and purchasing). Connectedness to nature was the factor most associated with the pro-environment behaviors (conservation, citizenship, purchasing). Moreover, newly we showed that attitudes toward exploration were associated with citizenship and purchasing behaviors. Overall, the results newly highlighted the importance of environment-related characteristics alongside general personality traits. Fostering environmental-related personality factors, such as connection to nature and attitudes towards exploration, may drive positive environmental action, suggesting novel approaches to build a more sustainable society.

## 1 Introduction

Human society is causing climate change and ecological damages and must act (Stollberg and Jonas, 2021). Individual pro-environmental behaviors that refer to various human actions that produce environmental benefits relative to other alternative behaviors (Lange and Dewitte, 2019) play a crucial role in addressing this issue. Among the pro-environmental behaviors, there are both public-sphere behaviors (such as environmental activism and nonactivist citizenships) and private-sphere behaviors (Stern, 2000). Within the latter, those with greatest impact include conservation of resources (e.g., heating, freshwater consumption), food and purchasing behaviors, and transportation decisions (Markle, 2013; Stern, 2000). These behaviors are typically studied collectively rather than individually, so their specific qualities may be overlooked. In this regard, understanding the factors that drive individuals to engage or refrain from pro-environmental behaviors is crucial. Research on these factors has used two approaches: a context-focused approach, exploring environmental factors on behaviors, and a person-oriented approach (Schultz and Kaiser, 2012), which emphasizes the importance of individual factors that encourage ecological behaviors.

Research has shown the importance of psychological and motivational factors, such as values, norms and internal attributions, in relation to pro-environmental behaviors (Bamberg and Möser, 2007; Stern, 2000). However, personality traits—defined as the individual typical ways of thoughts, feelings, and behaviors—seem also related to shaping environmental attitudes, values, and behaviors (Stern, 2000). Studies typically have shown a positive correlation between pro-environmental self-reported behaviors and openness, agreeableness and conscientiousness (e.g., Brick and Lewis, 2016; Markowitz and Shariff, 2012; Poškus and Žukauskienė, 2017; Soutter et al., 2020). Extraversion results are mixed, and no association has been found for neuroticism (e.g., Brick and Lewis, 2016; Soutter et al., 2020).

However, apart from personality traits, other personal characteristics may play a role. Some evidence underlines the importance of the characteristics related to an individual’s relationship with the environment, particularly with the concept of connectedness to nature and wayfinding inclinations. Connectedness to nature refers to a nature-based disposition of being bonded with the natural world, understanding its significance and value, and appreciating its beauty and benefits (Mayer and Frantz, 2004). Numerous studies have highlighted that individuals with a strong connection to nature are more inclined to engage in environmentally conscious actions (e.g., Martin et al., 2020). A recent meta-analysis has quantified this relationship as having a moderate effect size (r = 0.42; Whitburn et al., 2020). Moreover, this association remains consistent across gender, age groups and geographic locations (Whitburn et al., 2020).

Besides connectedness to nature, individual wayfinding inclinations are factors pertaining to one’s relationship with the environment. They are people’s attitudes toward navigating and exploring environments. Typically, they involve perceived proficiency in efficiently moving through the environment (sense of direction), pleasure in exploring places, and levels of spatial anxiety (Muffato et al., 2022). These inclinations can be considered spatial-based personal dispositions given that they tend to remain relatively stable throughout an individual’s adulthood (Muffato et al., 2023), even with a degree of malleability (He and Hegarty, 2020). To date, researchers have not, to our knowledge, investigated the relationship among wayfinding inclinations, connectedness to nature, and personality traits together in relation to various pro-environmental behaviors.

Indeed, individuals who exhibit a profile characterized by a strong environment-individual relationship (i.e., strong connection to nature, high pleasure in exploration, and low spatial anxiety) could be more likely to engage in pro-environmental behaviors. Therefore, we newly aimed to investigate the relationship between individual disposition, including general dispositions (personality traits) and specific environment-related dispositions, and various key pro-environmental behaviors (Kaiser, 2020), including conservation of resources, citizenship, food, and purchasing and transportation behaviors. This approach emphasizes specific associations between individual traits and behaviors. Although numerous studies have linked personality traits to pro-environmental behaviors (without necessarily examining various types of them), limited evidence exists regarding the conjoint role of environment-based dispositions. General and environment-based dispositions may play distinct roles, varying in relation to various pro-environmental behaviors.

We expect, concerning personality traits, the involvement of openness, agreeableness, conscientiousness, and extraversion, as research has shown (e.g., Brick and Lewis, 2016). However, openness, which consistently correlates with pro-environmental behaviors (e.g., Soutter et al., 2020), could be the factor with a stronger relationship with all the behaviors under investigation. Conscientiousness may be more closely associated with conservation behaviors (Brick and Lewis, 2016; Milfont and Sibley, 2012) and purchasing choices (Novliadi et al., 2018) given these pro-environmental behaviors need consistency and discipline. Agreeableness and extraversion may be more relevant for citizenship behaviors because individuals who are altruistic, outgoing, and social could tend to be more engaged in activities in the public sphere (Koole et al., 2001).

Concerning personal factors related to the individual-environment relationship, we expect to confirm a positive correlation between connectedness with nature and pro-environmental behaviors (Whitburn et al., 2020). Specifically, we may expect that connectedness to nature could be associated with not only conservation and purchasing behaviors as previously found (Martin et al., 2020), but also with the other domains of pro-environment behaviors given it is a specific environmental-related personal characteristic (Whitburn et al., 2020). Regarding the attitude toward exploring the environment, although no previous literature is available, we might expect individuals with lower spatial anxiety and who find greater pleasure in exploring could be more inclined to engage in citizenship behaviors and choose environmentally friendly transportation methods, such as walking, given they likely enjoy going outside more (Muffato et al., 2022).

To sum up, we can expect that each pro-environmental behavior will be primary associated with some of the individual factors, with environmental-related dispositions playing a role in these behaviors. However, we expect food choices to be a distinct factor less influenced by these personality characteristics (Spence, 2022), due to its potentially stronger connection to other (e.g., cultural and economic) factors.

To accomplish our goals, we conducted a self-reported study on various types of pro-environmental behaviors and assessed individual factors. Self-report assessments, although they have limitations regarding their reliability due to potential bias in individuals’ observations of their own behavior, offer a convenient way to capture various pro-environmental behavioral domains as well as their frequency (Tam and Chan, 2017) and timeframes. Items on self-report questionnaires may refer to the present, a specific period in the past (e.g., the previous month or year), or an unspecified timeframe (Lange and Dewitte, 2019). We opted to use an unspecified timeframe to capture usual individual behavior regarding conservation of resources, citizenship, food, purchasing, and transportation behaviors (using an adapted version of the Pro-environmental Behaviors Scale, Markle, 2013). Personality traits have been assessed with the Italian 44-item BIG-5 Inventory (BFI) (Ubbiali et al., 2013), connectedness to nature with the scale by Mayer and Frantz (2004), and attitudes toward exploration and spatial anxiety with the scales by De Beni et al. (2014).

## 2 Method

### 2.1 Participants

The study included 649 adults (405 women; 239 males; 5 other/prefer not to say) 18 to 59 years old (women: M age = 29.70, SD = 12.20; men: M age = 31.60, SD = 11.90; other/prefer not to say: M age = 28.00, SD = 9.03) recruited from a university course in exchange for course credit and by word of mouth. Inclusion criteria were Italian mother tongue; age between 18 and 59 years; and no history of psychiatric, neurological diseases, or diseases capable of causing cognitive, visual, auditory and/or motor impairments. We determined the sample size considering at least five observations for each parameter estimated in the model (Bollen, 1989); therefore, a total of at least 335 participants was sufficient (67 parameters in the model; see results).

### 2.2 Materials

#### 2.2.1 Pro-environmental behavior measure: revised version of the Italian version of the pro-environmental behavioral scale

The PEBS (Menardo et al., 2020; Markle, 2013; see Supplementary materials) consists of 15 items, evaluating environmentally favorable behaviors grouped into four factors: conservation (e.g., “How often do you cut down on heating or air conditioning to limit energy use?”); environmental citizenship (e.g., “How frequently do you watch television programs, movies, or internet videos about environmental issues?”); food (e.g., “How often do you consume beef?”), and transportation (e.g., “How often have you walked or cycled instead of driving?”). In addition to the original questionnaire for this study, we considered a fifth factor, purchasing (given it is another key pro-environment behavior), creating 4 items (inspired by from Kaiser, 2020; e.g., “How often do you prefer to repair used items instead of replacing them with new ones?”). We asked participants to rate how often they exhibit each behavior on a 5-point Likert scale (1 = never to 5 = always, except for 3 items; see Supplementary materials). Unlike Menardo et al. (2020), we did not provide a specific timeframe for responses about food because data collection occurred immediately after the COVID-19 pandemic, potentially impacting habits. To investigate the factorial structure of the new version of the questionnaire, we conducted a factor analysis to compare the five-factor structure (conservation, citizenship, food, transportation, and purchasing) with a single factor (all items loading a single pro-environmental latent variable). The results showed that the five-factor structure showed better fit indices (RMSEA = 0.037, SRMR = 0.048, CFI = 0.946, NNFI = 0.930, AIC = 34,402) than the one-factor structure (RMSEA = 0.098, SRMR = 0.077, CFI = 0.571, NNFI = 0.518, AIC = 352,044). Therefore, for the scores, we considered the five factors, each total score being the mean of the scores on the corresponding items. Reliability was moderate in the current sample (Cronbach’s alpha: conservation = 0.63, citizenship = 0.48, food = 0.68, transportation = 0.39, purchasing = 0.60; Omega: conservation = 0.63, citizenship = 0.40, food = 0.72, transportation = 0.38, purchasing = 0.73), reliability in the original version was good (Cronbach’s alpha range.62–0.74, Omega range 0.69–0.80).

#### 2.2.2 BFI, Italian version

The BFI (Ubbiali et al., 2013), consisting of 44 items, was used to assess the five personality traits: extraversion (8 items; example: “is outgoing, sociable”), agreeableness (9 items; example: “is considerate and kind to almost everyone”), conscientiousness (9 items; example: “does a thorough job”), neuroticism (8 items; example: “gets nervous easily”), and openness to experience (10 items; example: “is inventive”). The participant’s task was to indicate how much they agree with each statement on a 5-point Likert scale (from 1 = not at all to 5 = very much). The score is the sum of the items for each factor after we reverse the negative items. Cronbach’s alpha: extraversion = 0.85, agreeableness = 0.66, conscientiousness = 0.83, neuroticism = 0.80, openness = 0.79.

#### 2.2.3 Connectedness to nature scale

The CNS (Mayer and Frantz, 2004) questionnaire, consisting of 14 items, was used to assess one’s level of connection to nature and the environment, awareness of the connection between one’s well-being and the natural world, and ideas, attitudes, and emotional commitment regarding living things (fauna, flora). An example is “I recognize and value the intelligence of other living organisms.” Participants expressed agreement on a 5-point Likert scale (from 1 = strongly disagree to 5 = strongly agree). The total score is the sum of the item ratings. Cronbach’s alpha = 0.76.

#### 2.2.4 Wayfinding inclination measures

Attitudes toward orientation tasks questionnaire, the AtOT (De Beni et al., 2014). The questionnaire, consisting of 10 items, was used to assess a person’s attitudes toward exploring environments. An example is “I like to find new ways to reach familiar places.” Participants answered on 6-point Likert scale (from 1 = not at all to 6 = very much). The total score is the sum of the items. Cronbach’s alpha = 0.82. *Spatial Anxiety Questionnaire* (SA; De Beni et al., 2014). The questionnaire, consisting of 8 items, was used to assess the level of anxiety and discomfort one experiences when moving through space and in spatial situations. An example is “Park your automobile in a big parking area.” Participants answered on a 6-point Likert scale (from 1 = very little to 6 = very much). The total score is the sum of the items. Cronbach’s alpha = 0.92.

#### 2.2.5 Procedures

The study was conducted (in 2022) online using Qualtrics and Zoom in an individual session lasting around 30 min. The experimenter met the participant in a Zoom meeting and provided a Qualtrics link for them to complete independently. Participants read and signed the informed consent form, provided demographic information (age, gender) and, in random order, completed the revised version of the PEBS, the BFI, the CNS, the AtOT, and the SA. The experimenter remained available on Zoom to answer any questions participants had.

## 3 Results

We conducted analyses using R. At the descriptive level, we computed means and standard deviations of all considered variables and correlations between them. See Table 1.

**Table 1:** Descriptives and correlations between variables.

	M(SD)	1	2	3	4	5	6	7	8	9	10	11	12	13
1. Age	29.69 (11.14)													
2. BIG5-Conscientiousness	3.75 (0.71)	**0.29**												
3. BIG5-Openness	3.73 (0.62)	−0.10	0.03											
4. BIG5-Neuroticism	3.26 (0.75)	**−0.21**	**−0.29**	−0.07										
5.BIG5-Extraversion	3.23 (0.77)	0.07	**0.17**	**0.21**	**−0.32**									
6. BIG5-Agreebleness	3.75 (0.71)	**0.20**	**0.21**	**0.18**	**−0.20**	**0.25**								
7. Connectedness to nature	56.3 (8.30)	0.10	0.06	**0.26**	−0.06	0.02	**0.16**							
8. Attitude toward exploring	39.7 (8.61)	0.09	0.14	**0.24**	**−0.38**	**0.23**	0.11	0.11						
9. Spatial anxiety	22.0 (8.26)	−0.10	−0.12	−0.07	**0.37**	**−0.22**	−0.09	0.01	**−0.50**					
10. PEBS-Conservation	3.54 (0.74)	−0.08	0.06	**0.16**	0.05	−0.05	0.08	0.**24**	0.06	−0.04				
11. PEBS-Environmental citizenship	1.98 (0.58)	−0.01	−0.04	**0.20**	0.03	0.05	0.04	**0.26**	0.15	0.02	**0.25**			
12. PEBS-Food	3.12 (0.69)	0.00	0.02	**0.15**	0.10	−0.02	0.11	0.12	0.03	0.04	**0.27**	**0.20**		
13. PEBS-Transportation	2.88 (0.88)	**−0.16**	−0.14	**0.18**	−0.08	0.04	−0.04	0.06	**0.16**	−0.07	**0.20**	**0.17**	0.03	
14. PEBS-Purchasing	2.87 (0.78)	−0.02	0.02	**0.27**	0.06	0.02	0.06	**0.28**	**0.15**	0.00	**0.46**	**0.34**	**0.38**	**0.26**

*N = 649. Given multiple comparisons, only correlations with r ≥ |0.15|, p < 0.001 are considered significant (in bold type).*|N = 649. Given multiple comparisons, only correlations with r ≥ |0.15|, p < 0.001 are considered significant (in bold type).|, *p* < 0.001 are considered significant (in bold type).

**Figure 1:**
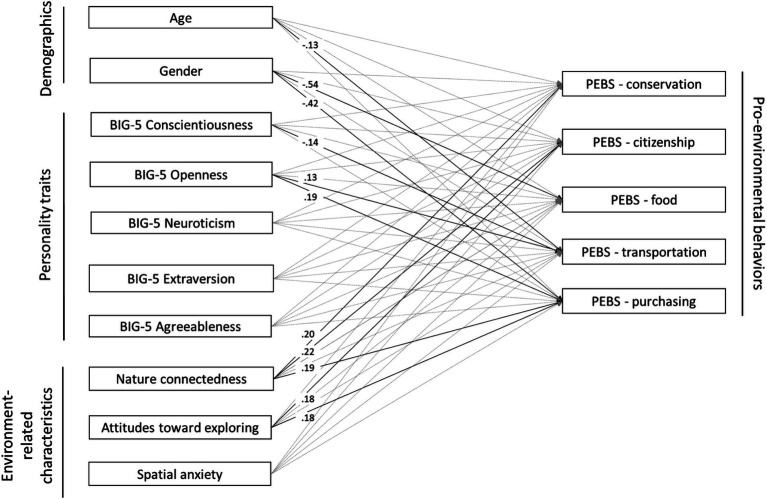
Graphical representation of the significant relationships (*p* ≤ 0.001) in the multivariate regression model. Covariances are present in the model but not shown in the figure.

Then, we ran a multivariate regression model to investigate the associations of personality traits (Big-5), connectedness to nature (CNS), and wayfinding inclinations (attitudes toward exploring places and spatial anxiety) in each domain of pro-environmental behavior, considering the covariance between them (full model; see Figure 1). We included age and gender in the model as a control given their potential relationship with pro-environmental behaviors (e.g., Gifford and Nilsson, 2014). Table 2 presents standardized betas, CI, *p* values (we considered *p* values ≤0.001 significant given the multiple comparisons), and R^2^ of the model. The results showed different predictors for the various pro-environmental behavior domains. Higher openness predicted greener transportation and purchasing behaviors (however, higher conscientiousness predicted less green transportation behavior). Higher connectedness to nature predicted greater conservation and citizenship as well as purchasing behaviors. More positive attitudes toward exploring predicted greener citizenship and purchasing behaviors. Concerning gender and age, women reported more green food and purchasing behaviors than men (but equal to “other/prefer not to say”); younger people reported greener transportation behaviors.

**Table 2:** Results of the multivariate regression analysis.

		Std β	CI lower	CI upper	*p*
Regression on PEBS-conservation (total R^2^ = 0.11)					
Demographics (R^2^ = 0.03)	Age	−0.10	−0.18	−0.02	0.010
	Gender	−0.25	−0.41	−0.10	0.002
Personality traits (R^2^ = 0.04)	BIG-5 Conscientiousness	0.07	−0.01	0.15	0.106
	BIG-5 Openness	0.09	0.01	0.17	0.031
	BIG-5 Neuroticism	0.07	−0.02	0.16	0.114
	BIG-5 Extraversion	−0.10	−0.18	−0.02	0.013
	BIG-5 Agreeableness	0.06	−0.02	0.14	0.149
Environment-related dispositions (R^2^ = 0.04)	**Nature connectedness**	**0.20**	**0.12**	**0.28**	**<0.0001**
	Attitude to explore	0.04	−0.05	0.13	0.330
	Spatial anxiety	−0.09	−0.17	0.00	0.049
Regression on PEBS-citizenship (total R^2^ = 0.11)					
Demographics (R^2^ = 0.00)	Age	0.01	−0.07	0.08	0.880
	Gender	−0.02	−0.18	0.13	0.766
Personality traits (R^2^ = 0.05)	BIG-5 Conscientiousness	−0.05	−0.13	0.03	0.195
	BIG-5 Openness	0.11	0.03	0.19	0.009
	BIG-5 Neuroticism	0.09	0.00	0.18	0.050
	BIG-5 Extraversion	0.04	−0.04	0.12	0.366
	BIG-5 Agreeableness	−0.01	−0.09	0.07	0.782
Environment-related dispositions (R^2^ = 0.06)	**Nature connectedness**	**0.22**	**0.14**	**0.30**	**<0.0001**
	**Attitude to explore**	**0.18**	**0.09**	**0.27**	**<0.0001**
	Spatial anxiety	0.08	−0.01	0.16	0.083
Regression on PEBS-food (total R^2^ = 0.12)					
Demographics (R^2^ = 0.07)	Age	0.03	−0.05	0.11	0.474
	**Gender**	**−0.54**	**−0.70**	**−0.38**	**<0.0001**
Personality traits (R^2^ = 0.04)	BIG-5 Conscientiousness	−0.02	−0.10	0.06	0.562
	BIG-5 Openness	0.12	0.04	0.20	0.003
	BIG-5 Neuroticism	0.08	−0.01	0.17	0.072
	BIG-5 Extraversion	−0.07	−0.15	0.01	0.072
	BIG-5 Agreeableness	0.08	0.00	0.16	0.048
Environment-related dispositions (R^2^ = 0.01)	Nature connectedness	0.04	−0.04	0.12	0.294
	Attitude to explore	0.10	0.01	0.19	0.037
	Spatial anxiety	0.00	−0.09	0.09	0.979
Regression on PEBS-transportation (total R^2^ = 0.10)					
Demographics (R^2^ = 0.03)	**Age**	**−0.13**	**−0.21**	**−0.05**	**0.001**
	Gender	−0.05	−0.21	0.11	0.550
Personality traits (R^2^ = 0.05)	**BIG-5 Conscientiousness**	**−0.14**	**−0.22**	**−0.06**	**0.001**
	**BIG-5 Openness**	**0.13**	**0.05**	**0.21**	**0.001**
	BIG-5 Neuroticism	−0.10	−0.19	−0.01	0.026
	BIG-5 Extraversion	−0.01	−0.09	0.08	0.893
	BIG-5 Agreeableness	−0.04	−0.12	0.04	0.275
Environment-related dispositions (R^2^ = 0.02)	Nature connectedness	0.03	−0.05	0.11	0.461
	Attitude to explore	0.13	0.04	0.22	0.005
	Spatial anxiety	0.00	−0.09	0.09	0.992
Regression on PEBS-purchasing (total R^2^ = 0.18)					
Demographics (R^2^ = 0.05)	Age	0.00	−0.08	0.08	0.993
	**Gender**	**−0.42**	**−0.58**	**−0.27**	**<0.0001**
Personality traits (R^2^ = 0.07)	BIG-5 Conscientiousness	−0.02	−0.10	0.06	0.651
	**BIG-5 Openness**	**0.19**	**0.11**	**0.27**	**<0.0001**
	BIG-5 Neuroticism	0.08	−0.01	0.16	0.081
	BIG-5 Extraversion	−0.04	−0.12	0.03	0.264
	BIG-5 Agreeableness	−0.01	−0.08	0.07	0.830
Environment-related dispositions (R^2^ = 0.06)	**Nature connectedness**	**0.19**	**0.12**	**0.27**	**<0.0001**
	**Attitude to explore**	**0.18**	**0.09**	**0.27**	**<0.0001**
	Spatial anxiety	0.02	−0.06	0.10	0.655

Coefficients significant with *p* < 0.001 in bold type.

## 4 Discussion

We aimed to investigate the interplay between general personality traits and environment-specific personality characteristics, such as connection to nature, exploration attitudes, and spatial anxiety, in relation to various pro-environmental behavior domains (i.e., conservation, citizenship, food, transportation, and purchasing behaviors). The multivariate regression analyses showed specificity of relationships between each predictor considered and the various domain of pro-environmental behavior, providing new evidence, as we discuss in the following paragraph.

Concerning personality traits as assessed using the BFI (Ubbiali et al., 2013), we identified openness as the most significant trait for pro-environmental behavior, consistent with the literature (Soutter et al., 2020). Specifically, we newly observed this pattern primarily for greener transportation and purchasing behaviors, suggesting that openness, which involve being open to new experiences and ideas, seem particularly related to behaviors that involve trying new things. For example, individuals may be more inclined to change their habits, explore new transportation options, or try innovative methods, such as using websites to buy and resell clothing. Conversely, conscientiousness was newly specifically found to be associated with lower engagement in green transportation behavior, possibly reflecting the fact that conscientious people could be more resistant to changing their habits in favor of more sustainable ones. Overall, we confirmed the significant role of personality traits in pro-environmental behaviors (Brick and Lewis, 2016).

However, when considered all together, environment-related dispositions also played a role. Indeed, our results confirmed the significant role of connectedness to nature in promoting various pro-environmental behaviors. Higher levels of connectedness to nature were associated with increased conservation, citizenship, and purchasing behaviors, in line with previous research (Martin et al., 2020). The connection to nature appears to be a robust predictor of pro-environmental behaviors across multiple domains, emphasizing the need to nurture this trait from a young age (Spano et al., 2021).

Finally, we introduced a novel environment-related dimension by examining the role of wayfinding inclinations, such as attitudes toward exploring places and spatial anxiety, on pro-environmental behaviors. The results newly suggest that attitudes toward exploration can shape pro-environmental actions. Specifically, we found an association with citizenship behaviors, possibly indicating that individuals who are more willing to explore their environment are more likely to engage in activities in the public sphere (Stern, 2000). Surprisingly, we also found an association with purchasing behavior, suggesting that individuals with favorable attitudes toward exploration may be more open to exploring new purchasing methods. We also expected an association with transportation behavior (Muffato et al., 2022) but did not find one. This could be attributed to the significant cost implications associated with changing transportation habits. For instance, transitioning from non-sustainable to sustainable transportation often requires more time (Mouratidis et al., 2023), an effect that may be stronger than the role of individual characteristics in this context. Researchers should explore this intriguing connection to understand its potential better. This personal attitude seems specific, not completely overlapping with general personality traits, such as openness. It also differs from other wayfinding inclinations, such as spatial anxiety, in its ability to predict certain pro-environment behaviors. This suggests the importance of promoting positive attitudes toward exploration because the experience of exploration may be important for positive behaviors related to the environment.

Lastly, we included age and gender as controls, given their relevance to pro-environment behaviors. Women reported higher engagement in green food and purchasing behaviors than men (e.g., Gifford and Nilsson, 2014). Younger individuals were more likely to choose greener transportation options than older ones, possibly reflecting either higher sensitivity to environmental issues or limited car access. Nevertheless, demographic variables are important in understanding pro-environmental behaviors.

The present results offer insights into practical applications, such as promoting both general and specific environmentally related dispositions. For instance, traits like conscientiousness and openness to experiences can change in response to environmental influences (e.g., Roberts and Bogg, 2004; Schwaba et al., 2018), as can connectedness to nature (e.g., Coughlan et al., 2022) and attitudes toward exploration (Meneghetti et al., 2019). Exposure to intentional activities and specific environments can therefore support changes in individual dispositions and, in turn, increase pro-environmental behaviors.

However, some limitations need to be considered and possibly addressed in future research. The present study is a correlational study based only on self-reported measures. Using objective measures of pro-environmental behaviors (e.g., Lange and Dewitte, 2019) and conducting a longitudinal study would provide stronger evidence of the association between personal characteristics and various pro-environmental behaviors. It is important to note that although we used a well-known and validated scale from the literature (Markle, 2013) and adapted it for the Italian context (Menardo et al., 2020) to measure pro-environmental behaviors, the measure in the present sample did not show high reliability, especially for the citizenship and transportation factors. This limitation could have impacted the results found. More research is needed to create valid instruments. Furthermore, other personal characteristics should be explored more thoroughly in each pro-environmental domain, for instance socio-demographic aspects, perceived costs and attitudes towards environmental governance (Fischer, 2010; Kaplowitz and Boucher, 2022). Additionally, generalizability of the association found is not possible, and replications of this study in other countries should be conducted. Another limitation is the lack of a social desirability scale, which could have helped minimize potential bias. Finally, as expected, we found that food behaviors were not associated with general or environment-specific personality traits. This suggests that future research should pay more attention to the specificity of each environmental behavior per se.

To conclude, the present study offers a fresh perspective on the individual factors relating to pro-environmental behaviors in various domains. We emphasized that specific environmental-related personal characteristics, and not only general personality traits, play a role in driving pro-environmental behaviors. Each pro-environmental behavior can have specific associations with both general and specific environmental dispositions. Newly, we found that individual attitudes toward exploration associated with certain pro-environmental behaviors, suggesting the importance of the relationship between the individual and the environment for at least some of the pro-environmental behaviors. Therefore, acknowledging the multifaceted nature of pro-environmental actions, our results underscore the significance of promoting individual characteristics related to the environment to facilitate the creation of a more sustainable society.

## Data Availability

The datasets presented in this study can be found in online repositories. The names of the repository/repositories and accession number(s) can be found at Figshare doi: 10.6084/m9.figshare.24511318.
