# Supplementary material for: Are explorers greener? Investigating the role of personality traits, connectedness to nature and attitudes toward exploring in various pro-environmental behaviors
Source: Front Psychol. 2025 Jan 15;15:1404095. doi: 10.3389/fpsyg.2024.1404095 (PMC11774957; doi:10.3389/fpsyg.2024.1404095)
Supplement: Supplementary file 1 [file Table_1.docx]

|  |  | **Covariance** |  |  |  |
| --- | --- | --- | --- | --- | --- |
|  | Conservation ~~ Citizenship | .17 |  |  |  |
|  | Conservation ~~ Food | .18 |  |  |  |
|  | Conservation ~~ Transportation | .16 |  |  |  |
|  | Conservation ~~ Purchasing | .33 |  |  |  |
|  | Citizenship ~~ Food | .14 |  |  |  |
|  | Citizenship ~~ Transportation | .11 |  |  |  |
|  | Citizenship ~~ Purchasing | .22 |  |  |  |
|  | Food ~~ Transportation | .02 |  |  |  |
|  | Food ~~ Purchasing | .26 |  |  |  |
|  | Transportation ~~ Purchasing | .20 |  |  |  |
|  | Age ~~ Gender | .02 |  |  |  |
|  | Age ~~ Big-5 consciousness | .29 |  |  |  |
|  | Age ~~ Big-5 openness | -.10 |  |  |  |
|  | Age ~~ Big-5 Neuroticism | -.21 |  |  |  |
|  | Age ~~ Big-5 extraversion | .07 |  |  |  |
|  | Age ~~ Big-5 agreeableness | .20 |  |  |  |
|  | Age ~~ Nature connectedness | .10 |  |  |  |
|  | Age ~~ Attitude to explore | .09 |  |  |  |
|  | Age ~~ Spatial anxiety | -.10 |  |  |  |
|  | Gender ~~ Big-5 consciousness | -.06 |  |  |  |
|  | Gender ~~ Big-5 openness | -.01 |  |  |  |
|  | Gender ~~ Big-5 neuroticism | -.11 |  |  |  |
|  | Gender ~~ Big-5 extraversion | -.02 |  |  |  |
|  | Gender ~~ Big-5 agreeableness | -.04 |  |  |  |
|  | Gender ~~ Nature connectedness | -.05 |  |  |  |
|  | Gender ~~ Attitude to explore | .11 |  |  |  |
|  | Gender ~~ Spatial anxiety | -.11 |  |  |  |
|  | Big-5 consciousness ~~ Big-5 openness | .03 |  |  |  |
|  | Big-5 consciousness ~~ Big-5 neuroticism | -.29 |  |  |  |
|  | Big-5 consciousness ~~ Big-5 extraversion | .17 |  |  |  |
|  | Big-5 consciousness ~~ Big-5 agreeableness | .21 |  |  |  |
|  | Big-5 consciousness ~~ Nature connectedness | .06 |  |  |  |
|  | Big-5 consciousness ~~ Attitude to explore | .14 |  |  |  |
|  | ~~ Spatial anxiety | -.12 |  |  |  |
|  | Big-5 openness ~~ Big-5 neuroticism | -.07 |  |  |  |
|  | Big-5 openness ~~ Big-5 extraversion | .21 |  |  |  |
|  | Big-5 openness ~~ Big-5 agreeableness | .18 |  |  |  |
|  | Big-5 openness ~~ Nature connectedness | .26 |  |  |  |
|  | Big-5 openness ~~ Attitude to explore | .24 |  |  |  |
|  | Big-5 openness ~~ Spatial anxiety | -.07 |  |  |  |
|  | Big-5 neuroticism ~~ Big-5 extraversion | -.32 |  |  |  |
|  | Big-5 neuroticism ~~ Big-5 agreeableness | -.20 |  |  |  |
|  | Big-5 neuroticism ~~ Nature connectedness | -.06 |  |  |  |
|  | Big-5 neuroticism ~~ Attitude to explore | -.38 |  |  |  |
|  | Big-5 neuroticism ~~ Spatial anxiety | .37 |  |  |  |
|  | Big-5 extraversion ~~ Big-5 agreeableness | .25 |  |  |  |
|  | Big-5 extraversion ~~ Nature connectedness | .02 |  |  |  |
|  | Big-5 extraversion ~~ Attitude to explore | .23 |  |  |  |
|  | Big-5 extraversion ~~ Spatial anxiety | -.22 |  |  |  |
|  | Big-5 agreeableness ~~ Nature connectedness | .16 |  |  |  |
|  | Big-5 agreeableness ~~ Attitude to explore | .11 |  |  |  |
|  | Big-5 agreeableness ~~ Spatial anxiety | -.09 |  |  |  |
|  | Nature connectedness ~~ Attitude to explore | .11 |  |  |  |
|  | Nature connectedness ~~ Spatial anxiety | .01 |  |  |  |
|  | Attitude to explore ~~ Spatial anxiety | -.50 |  |  |  |

*Note.* Coefficients significant with *p* < .001 in bold type.
