# Supplementary material for: Are explorers greener? Investigating the role of personality traits, connectedness to nature and attitudes toward exploring in various pro-environmental behaviors
Source: Front Psychol. 2025 Jan 15;15:1404095. doi: 10.3389/fpsyg.2024.1404095 (PMC11774957; doi:10.3389/fpsyg.2024.1404095)
Supplement: Supplementary file 2 [file Data_Sheet_1.docx]

Supplementary Material

# Revised version of the Italian Pro-Environmental Behavioural scale (adapted from Menardo et al., 2020; Markle, 2013)

| From Menardo et al., 2020 | Conservation | 1. Quanto spesso spegni le modalità standby di elettrodomestici o dispositivi elettronici?  1= mai; 2= raramente; 3= a volte; 4= solitamente; 5= sempre |
| --- | --- | --- |
| From Menardo et al., 2020 | Conservation | 2. Quanto spesso riduci il riscaldamento o l'aria condizionata per limitare il consumo di energia?  1= mai; 2= raramente; 3= a volte; 4= solitamente; 5= sempre |
| From Menardo et al., 2020 | Conservation | 3. Quanto spesso limiti il tuo tempo sotto la doccia per risparmiare acqua?  1= mai; 2= raramente; 3= a volte; 4= solitamente; 5= sempre |
| From Menardo et al., 2020 | Conservation | 4. Quanto spesso aspetti di avere un carico completo per usare la lavatrice o la lavastoviglie?  1= mai; 2= raramente; 3= a volte; 4= solitamente; 5= sempre |
| From Menardo et al., 2020 | Citizenship | 5. Sei attualmente membro di un gruppo ambientale, di conservazione o di protezione della fauna selvatica?  1= no; 5= si |
| From Menardo et al., 2020 | Citizenship | 6. Durante l'ultimo anno hai contribuito con del denaro a gruppi di tutela ambientale, di conservazione o protezione della fauna selvatica?  1= no; 5= si |
| From Menardo et al., 2020 | Citizenship | 7. Con quale frequenza guardi programmi televisivi, film o video su Internet sui problemi ambientali?  1= mai; 2= raramente; 3= a volte; 4= solitamente; 5= sempre |
| From Menardo et al., 2020 | Citizenship | 8. Quanto spesso parli con gli altri dei loro comportamenti pro-ambientali?  1= mai; 2= raramente; 3= a volte; 4= solitamente; 5= sempre |
| Adapted from Menardo et al., 2020** | Food | 9. Quanto spesso consumi frutta e verdura coltivata a basso impatto ambientale (ad es. km 0, biologica)?  1= mai; 2= raramente; 3= a volte; 4= solitamente; 5= sempre |
| Adapted from Menardo et al., 2020** | Transportation | 10. Rispondi alla seguente domanda in base al veicolo a motore che usi più spesso: approssimativamente quanti chilometri al litro fa il veicolo?  1 = non lo so o 10 o meno; 2 = 11-12 ; 3 = 13-14; 4 = 15-16; 5 = 17 o più; 6 = non mi sposto con veicoli a motore |
| Adapted from Menardo et al., 2020** | Food | 11. Quanto spesso consumi carne di manzo?  1= mai; 2= raramente; 3= a volte; 4= solitamente; 5= sempre |
| Adapted from Menardo et al., 2020 | Food | 12. Quanto spesso consumi carne di maiale?  1= mai; 2= raramente; 3= a volte; 4= solitamente; 5= sempre |
| Adapted from Menardo et al., 2020** | Food | 13. Quanto spesso consumi pollame?  1= mai; 2= raramente; 3= a volte; 4= solitamente; 5= sempre |
| From Menardo et al., 2020 | Transportation | 14. Quanto spesso utilizzi i mezzi pubblici per spostarti?  1= mai; 2= raramente; 3= a volte; 4= solitamente; 5= sempre |
| From Menardo et al., 2020 | Transportation | 15. Quanto spesso cammini o pedali invece di utilizzare automobile o motorino?  1= mai; 2= raramente; 3= a volte; 4= solitamente; 5= sempre |
| New item* | Purchasing NEW | 16. Con quale frequenza presti attenzione nell’acquisto di prodotti con pochi imballaggi?  1= mai; 2= raramente; 3= a volte; 4= solitamente; 5= sempre |
| New item* | Purchasing | 17. Con quale frequenza presti attenzione alla provenienza dei prodotti che acquisti?  1= mai; 2= raramente; 3= a volte; 4= solitamente; 5= sempre |
| New item* | Purchasing | 18. Quanto spesso preferisci acquistare capi di abbigliamento di seconda mano rispetto che nuovi?  1= mai; 2= raramente; 3= a volte; 4= solitamente; 5= sempre |
| New item* | Purchasing | 19. Quanto spesso preferisci riparare oggetti usato rispetto al sostituirli con oggetti nuovi  1= mai; 2= raramente; 3= a volte; 4= solitamente; 5= sempre |

**Note**. The current scale: *includes a fifth factor related to purchasing behaviors and **for some of the items does not require respondents to specify a particular timeframe when providing their answers, unlike Menardo et al. (2020), and Markle (2013).

**
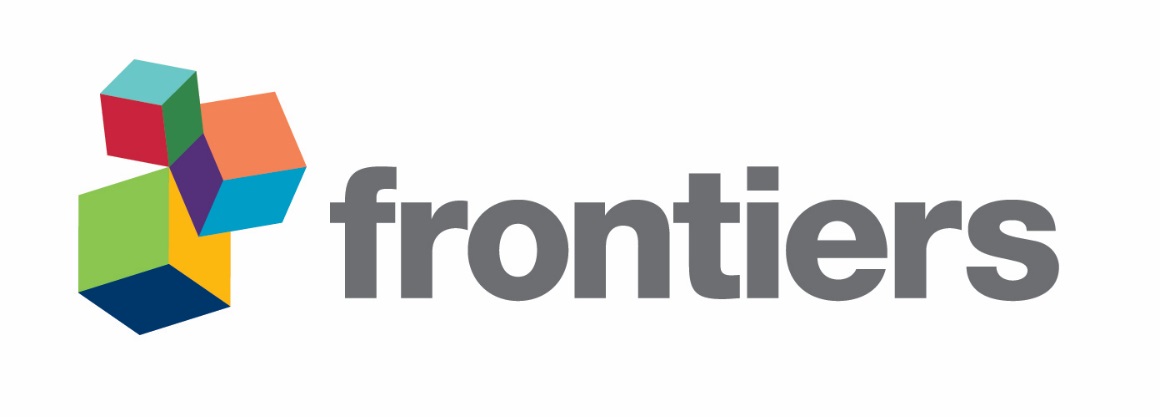
**
